# Secreted Frizzled-Related Protein 2 and Extracellular Volume Fraction in Patients with Heart Failure

**DOI:** 10.1155/2020/2563508

**Published:** 2020-05-06

**Authors:** Shaomin Yang, Haixiong Chen, Kuan Tan, Fusheng Cai, Yongxing Du, Weibiao Lv, Qiugen Hu, Yunzhao Hu, Yuli Huang

**Affiliations:** ^1^Department of Radiology, Shunde Hospital, Southern Medical University (The First People's Hospital of Shunde), Foshan, China; ^2^Department of Cardiology, Shunde Hospital, Southern Medical University, Foshan, China; ^3^Department of Laboratory Medicine, Shunde Hospital, Southern Medical University, Foshan, China

## Abstract

**Background:**

Quantification of extracellular volume (ECV) fraction by cardiovascular magnetic resonance (CMR) has emerged as a noninvasive diagnostic tool to assess myocardial fibrosis. Secreted frizzled-related protein 2 (SFRP2) appears to play an important role in cardiac fibrosis. We aimed to evaluate the association between SFRP2 and myocardial fibrosis and the prognostic value of ECV fraction in patients with heart failure (HF).

**Methods:**

In this prospective cohort study, 72 hospitalized adult patients (age ≥ 18 years) with severe decompensated HF were included. CMR measurements and T1 mapping were performed to calculate ECV fraction. Serum SFRP2 level was detected by an enzyme-linked immunosorbent assay kit. All patients were followed up, and the primary outcomes were composite events including all-cause mortality and HF hospitalization.

**Results:**

During the median follow-up of 12 months, 27 (37.5%) patients experienced primary outcome events and had higher levels of N-terminal pro-B-type natriuretic peptide (NT-proBNP), SFRP2, and ECV fraction compared with those without events. In Pearson correlation analysis, levels of SFRP2 (*r* = 0.33), high-sensitivity C-reactive protein (*r* = 0.31), and hemoglobin A1c (*r* = 0.29) were associated with ECV fraction (all *P* < 0.05); however, in multivariate linear regression analysis, SFRP2 was the only significant factor determined for ECV fraction (*r*_partial_ = 0.33, *P* = 0.02). In multivariate Cox regression analysis, age (each 10 years, hazard ratio (HR) 1.13, 95% confidence interval (CI) 1.04–1.22), ECV fraction (per doubling, HR 1.68, 95% CI 1.03–2.74), and NT-proBNP (per doubling, HR 2.46, 95% CI 1.05–5.76) were independent risk factors for primary outcomes.

**Conclusions:**

Higher ECV fraction is associated with worsened prognosis in HF. SFRP2 is an independent biomarker for myocardial fibrosis. Further studies are needed to explore the potential therapeutic value of SFRP2 in myocardial fibrosis.

## 1. Introduction

Heart failure (HF) is a growing global public health burden [1]. It is estimated that the prevalence of HF among the adult population is 1%–2%, but there are reports of proportions as high as 10% [2]. Myocardial fibrosis is a key pathological process in HF [3]. It predicts risk and represents a potential therapeutic target, and its measurement holds promise for future precision medicine [4]. Although myocardial biopsy is the gold standard for evaluating myocardial fibrosis, it is an invasive procedure with a high risk of complication. Recently, quantification of extracellular volume (ECV) fraction by T1-mapping technique in cardiovascular magnetic resonance (CMR) imaging has emerged as a novel, noninvasive diagnostic tool to assess myocardial fibrosis [5]. Studies have demonstrated the importance of myocardial fibrosis as estimated by CMR in different cohorts of patients [6]; however, there are limited data on the prognostic effect of ECV fraction in patients with advanced HF.

The wingless (Wnt) signaling pathway plays an important role in cardiac fibrosis [7]. A class of Wnt antagonist that has gained increasing attention as a potential serum biomarker and therapeutic target is the secreted frizzled-related protein (SFRP) family. Five members of the SFRP family (SFRP1–SFRP5) have been identified in mammals, among which SFRP2 is considered to be the most potent [8, 9]. Recent studies indicate that SFRP2 plays an important role in cardiac fibrosis, affecting multiple molecular pathways [10]. However, the results of basic research studies have been greatly inconsistent, showing both inhibition [11, 12] and promotion [13–16] of cardiac fibrosis in different research models. SFRP2 treatment can attenuate the adverse effects of doxorubicin-induced oxidative stress and apoptosis in muscle cells [17]. SFRP2 may also regulate the growth of cardiac fibroblasts and regulate cardiomyocyte energy metabolism and extracellular matrix remodeling [14]. These data indicated that SFRP2 may play a role in myocardial fibrosis and heart failure. However, there was no data on SFRP2 in patients with HF that has been reported currently.

In this study, we investigated the association between SFRP2 and myocardial fibrosis, as measured with CMR, among patients with advanced HF. We also explored the possibility that ECV fraction and SFRP2 could serve as new biomarkers for prognosis in HF.

## 2. Methods

### 2.1. Study Design and Population

In this prospective cohort study, hospitalized adult patients (age ≥ 18 years) with severe decompensated HF and New York Heart Association (NYHA) functional class III–IV were screened from January 2019 to January 2020. Decompensated HF was defined as new-onset HF or decompensation of chronic HF resulting in hospitalization and requiring treatment with intravenous diuretics, inotropic agents, or vasodilators [18].

Patients with contraindications to CMR (pacemaker or claustrophobia), acute myocardial infarction (MI), sepsis, history of malignancy, severe renal failure (estimated glomerular filtration rate (eGFR) < 30 mL/min/ 1.73 m^2^ or under renal replacement therapy), active bleeding or severe anemia (Hb < 60 g/L), autoimmune disease, or severe hepatic disease (bilirubin > 3× the upper limit of normal, or aspartate aminotransferase/alanine aminotransferase/alkaline phosphatase > 5× the upper limit of normal, or cirrhosis) were excluded.

All patients were treated in accordance with the principles recommended by the Chinese guidelines on HF [19]. The study complied with the principles of the Declaration of Helsinki and was approved by the central committee of the institutional review board at Shunde Hospital, Southern Medical University, China. Written informed consent was obtained from all participants.

### 2.2. Baseline Characteristics and SFRP2 Detection

Baseline characteristics including age, sex, smoking status, history of hypertension, diabetes mellitus (DM), cardiovascular disease, admission blood pressure (BP), heart rate, electrocardiogram, biochemistry tests, medicine, cardiac functional class, and echocardiographic parameters were collected from the hospital medical records. Hypertension was defined as systolic BP ≥ 140 mmHg and/or diastolic BP ≥ 90 mmHg, according to the current Chinese guidelines for the management of hypertension [20]. DM was defined as use of medications for diabetes or fasting blood glucose ≥ 7.0 mmol/L and/or hemoglobin A1c (HbA1_C_) ≥ 6.5% [21]. The eGFR was calculated by the Modification of Diet in Renal Disease equation adapted for Chinese patients [22]. Based on echocardiography, patients with left ventricular ejection fraction (LVEF) < 40%, 40%–49%, and ≥ 50% were defined as having HF with reduced ejection fraction (HFrEF), midrange EF (HFmrEF), and preserved EF (HFpEF), respectively [18]. Body mass index was calculated based on the height and weight collected on the day of CMR examination.

Fasting venous blood was collected and subjected to centrifugation at 1500 × *g* and 4°C for 15 min; serum was obtained and stored at −80°C until analysis. We used an enzyme-linked immunosorbent assay kit (R&D Systems, Minneapolis, MN, USA) to detect the levels of SFRP2, in accordance with the manufacturer's protocol. The intra-assay and interassay variations were 7.5% and 9.4%, respectively.

### 2.3. Cardiovascular Magnetic Resonance Examination

All the patients were scheduled for CMR examination when stabilized (without significant congestive symptom and sign) after treatment. CMR measurements and T1 mapping were performed by two experienced CMR radiologists. All patients were examined in the supine position using a 3.0 T scanner (Skrya; Siemens Medical Solutions, Erlangen, Germany). Briefly, after localization of the heart, to assess left ventricular (LV) and right ventricular (RV) myocardial function and mass, 10–12 consecutive short-axis images and 2-, 3-, and 4-chamber long-axis images of the LV were acquired using a steady-state free precession (SSFP) sequence. Then, midventricular short-axis modified Look-Locker inversion recovery (MOLLI) images were acquired for T1 determination using an 11-image, 18-heartbeat 3-(3)-3-(3)-5 SSFP sequence. A total dose of 0.1 mmol/kg gadobutrol (Gadavist, Bayer Healthcare Leverkusen, Germany) was injected at a rate of 2.0–3.0 mL/s; 10–15 min after contrast injection, short- and long-axis 2D inversion recovery late gadolinium enhancement images were acquired to evaluate focal myocardial fibrosis. Finally, 15 min postcontrast, MOLLI T1 mapping was repeated in a protocol identical to that used for precontrast T1 mapping (TR/TE 275.52/1.06 ms, 8 mm thickness, field of view 360 × 300 mm^2^, matrix size 256 × 144, and flip angle 35°).

Pre- and postcontrast myocardial T1 were measured in six regions of interest (ROIs) in the myocardium (anterior, anterolateral, inferolateral, inferior, inferoseptal, and anteroseptal) and in the LV blood pool. We took special care to avoid partial-volume effects from neighboring tissues or blood pool when delineating the ROI. The following formula was used to calculate ECV fraction [23], in which R1 represents 1/T1, myo pre and myo post represent the pre- and postcontrast myocardial T1 values, respectively, and blood pre and blood post represent the pre- and postcontrast blood pool T1 values, respectively.

(1) $$\begin{array}{c} \text{ECV fraction}=\left(1-\text{hematocrit}\right)\times \frac{\text{R}1\text{ myo post}-\text{R}1\text{ myo pre}}{\;\text{R}1\text{ blood post}-\text{R}1\text{ blood pre}}. \end{array}$$

### 2.4. Follow-Up and Endpoint Ascertainment

Patients were followed by review of the electronic medical record and/or telephone interview with the participants (or their family members if deceased). The primary outcomes were defined as composite endpoints of all-cause death or HF rehospitalization, which was defined as rehospitalization with HF as the primary cause and requirement for treatment with intravenous inotropes, vasodilators, or diuretics [24].

### 2.5. Statistical Analysis

All HF patients were categorized as with or without primary events during follow-up. Continuous variables are presented as mean and standard deviation (SD) or median and interquartile range. Categorical variables are presented as number and percentage. Baseline characteristics of patients with or without primary events were compared using the chi-squared test for categorical variables, two-tailed *t*-test for normally distributed continuous variables, and Wilcoxon rank-sum test for nonnormally distributed continuous variables.

Non-Gaussian data, including N-terminal pro-B-type natriuretic peptide (NT-proBNP) level, LVEF, eGFR, high-sensitivity C-reactive protein (hs-CRP) level, ECV fraction, and SFRP2 level were log_2_-transformed. The Pearson product-moment correlation coefficient (*r*) was calculated as a measure of linear association between covariates and ECV fraction. Multivariate linear regression analysis was performed to identify factors associated with levels of ECV fraction. Multicollinearity was evaluated by calculating the variance inflation factor (VIF), with a VIF > 2.5 suggesting the presence of multicollinearity [25].

Receiver operating characteristic (ROC) curves were used to identify the potential relationships among ECV fraction, SFRP2 and NT-proBNP levels, and the primary outcomes. Patients were divided into the higher ECV fraction group, higher SFRP2 level group, higher NT-proBNP level group, and control group, according to their median levels, respectively. Kaplan–Meier curves were employed to evaluate the study endpoints over time, and the log-rank test was used to assess differences in outcomes between different marker groups. Predictors of outcome were analyzed through univariate and multivariate Cox regression analyses. Those risk factors with a *P* value < 0.10 in univariable associations were included in the multivariable model. Proportionality assumptions for the Cox regression models were evaluated by log-log survival curves with the use of Schoenfeld residuals.

Statistical analyses were performed using SAS Enterprise Guide version 7.1 (SAS Institute, Cary, NC, USA). All *P* values were two-tailed, and values of *P* < 0.05 were considered statistically significant.

## 3. Results

### 3.1. Baseline Characteristics

**Figure 1:**
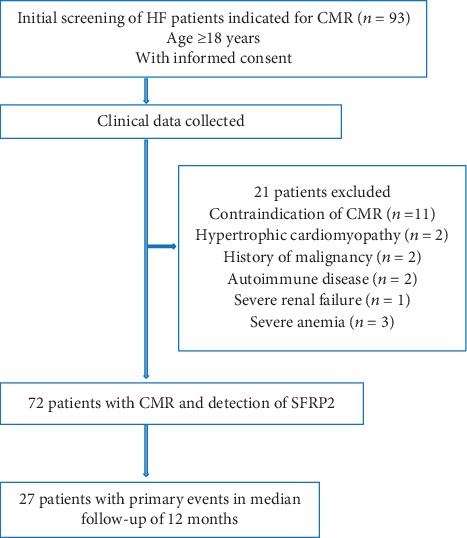
Flow chart of the study. CMR: cardiac magnetic resonance; HF: heart failure; SFRP2: secreted frizzled-related protein 2.

Of the 93 patients who were screened, 21 were excluded due to predefined exclusion criteria. Among the remaining 72 patients who were included in the analysis (Figure 1), 49 were male (68.1%) and 32 had diabetes (44.4%). Forty-six of them (63.9%) were with new-onset HF and 35 (36.1%) were with decompensation of chronic HF. Seventy patients were with congestive symptom and physical sign; only 2 patients were with volume redistribution. During the acute phase of HF, 70 patients with congestive symptom all received intravenous diuretics. Furthermore, 43 (59.7%), 15 (20.8%), and 2 (2.8%) patients received vasodilator, inotropes, and vasopressor treatment, respectively. Seven patients were with indication for implantable cardioverter defibrillator implantation for primary prevention of sudden death. Compared with patients without primary outcomes during follow-up, those with events had higher levels of NT-proBNP, SFRP2, and ECV fraction. Other demographic and clinical characteristics at admission were similar between the two groups (Table 1). Furthermore, clinical characteristics and medication at discharge were also not with significant difference between the two groups (Table 2).

### 3.2. Associations of ECV Fraction with Clinical and Laboratory Variables

Pearson product-moment correlation analysis showed that the clinical variables associated with ECV fraction, in decreasing order of strength of association, were as follows: SFRP2 (*r* = 0.33, *P* = 0.02), hs-CRP (*r* = 0.31, *P* = 0.02), and HbA1c (*r* = 0.29, *P* = 0.047) (Table 3). However, in multivariate linear regression analysis, SFRP2 was the only significant factor determined for ECV fraction (*r*_partial_ = 0.33, *P* = 0.02). The *R*^2^ value of SFRP2 for ECV fraction was 0.093, which indicated that 9.3% of the total ECV fraction variation can be attributed to the SFRP2 level. Multicollinearity was excluded because the VIF was 1.00.

### 3.3. Prognostic Value of Markers in HF Patients

**Figure 2:**
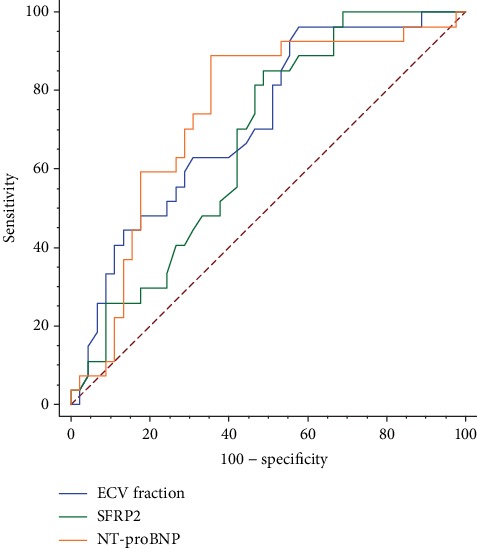
Comparisons between AUCs on ROCs. AUC: area under the curve; ECV: extracellular volume; NT-proBNP: N-terminal pro-B-type natriuretic peptide; ROC: receiver operating characteristics; SFRP2: secreted frizzled-related protein 2.

During a mean follow-up period of 12.2 months (interquartile 9.6–13.8 months), 27 (37.5%) patients in the cohort experienced the composite primary outcome, comprising 4 (5.6%) all-cause deaths and 23 (31.9%) HF hospitalizations. ROC analysis showed that levels of ECV fraction (area under the curve (AUC) 0.72, 95% confidence interval (CI) 0.60–0.82; *P* < 0.001), NT-proBNP (AUC 0.74, 95% CI 0.63–0.84; *P* < 0.001), and SFRP2 (AUC 0.67, 95% CI 0.55–0.78; *P* = 0.007) had predictive effects on the primary outcomes. Comparisons between AUCs showed that there were no significant differences among the three markers in ROC (Figure 2, all *P* > 0.05).

**Figure 3:**
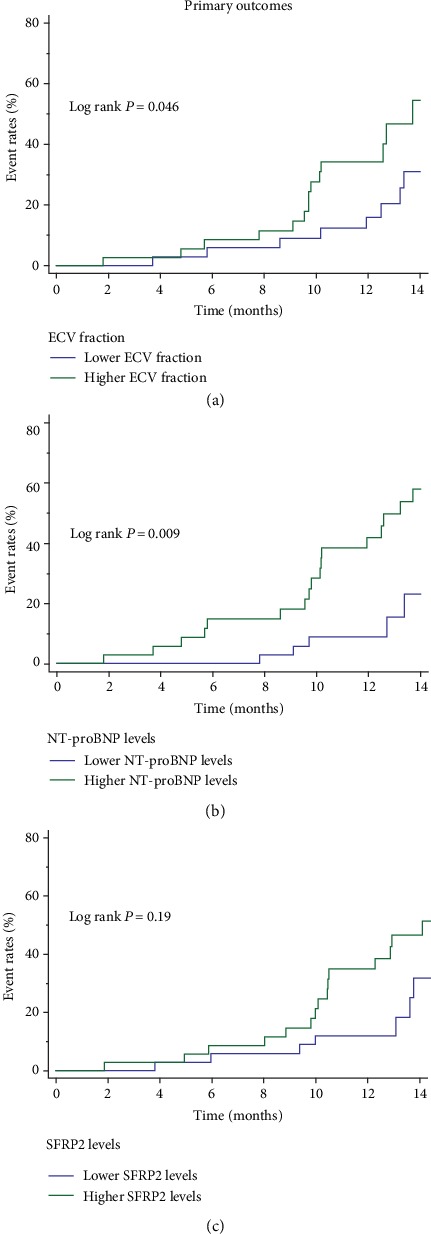
Cumulative incidence curves for the primary outcomes according to the different markers. (a) ECV fraction. (b) NT-proBNP. (c) SFRP2. ECV: extracellular volume; NT-proBNP: N-terminal pro-B type natriuretic peptide; ROC: receiver operating characteristics; SFRP2: secreted frizzled-related protein 2.

Kaplan–Meier survival analysis demonstrated that higher levels of ECV fraction (log-rank test: *P* = 0.046) and NT-proBNP (log-rank test: *P* = 0.009) were significant predictors of primary outcomes. However, the association was not significant in the higher SFRP2 group (log-rank test: *P* = 0.19) (Figure 3). In multivariate Cox regression models, age (each 10 years, hazard ratio (HR) 1.13, 95% CI 1.04–1.22), higher ECV fraction (per doubling, HR 1.68, 95% CI 1.03–2.74), and NT-proBNP (per doubling, HR 2.46, 95% CI 1.05–5.76) were independent risk factors for the primary outcomes (Table 4).

## 4. Discussion

This is the first study to report that serum SFRP2, an important Wnt signaling pathway modulator, is an independent marker of myocardial fibrosis. We also found that a higher ECV fraction was associated with worse prognosis in patients with severe HF, after adjusting for other risk factors.

The ECV fraction of T1 mapping is a noninvasive indicator of myocardial fibrosis that is highly coincident with myocardial biopsy results [26, 27]. The accumulation of excess type I collagen is the main change in myocardial fibrosis, and it results in expansion of the extracellular matrix (ECM). The ECV fraction obtained from native T1 and postcontrast T1 can measure expanded ECM, so the ECV fraction is assumed to reflect myocardial fibrosis when the myocardium is without edema or protein deposition. Because the ECV fraction combines the variation in both native and postcontrast T1 and measures the fraction of water volume occupied by the extracellular space in the myocardium, it may be more accurate than other CMR metrics for the evaluation of myocardial fibrosis [28]. Recently, Treibel et al. enrolled 1,714 consecutive patients without amyloidosis or hypertrophic cardiomyopathy from a single CMR referral center. Over a median follow-up of 5.6 years, ECV fraction exhibited more robust associations with outcomes than other surrogate CMR measures of myocardial fibrosis [4]. Furthermore, Roy et al. reported that, among HFpEF patients, high ECV fraction was associated with an increased risk of all-cause death and HF hospitalization in short-term follow-up [6]. Consistent with these previous studies, our study found that, in patients with severe HF (i.e., all cardiac functions at NYHA classes III–IV), higher ECV was associated with a worse outcome, further supporting the suggestion that ECV fraction should be evaluated in HF patients to access myocardial fibrosis and stratify risk.

We found it interesting that SFRP2 was significantly positively associated with the level of ECV fraction. Animal studies that focused on the effect of SFRP2 on myocardial fibrosis produced highly inconsistent results. Kobayashi et al. reported that collagen deposition was inhibited after MI in genetically modified *Sfrp2*-null mice compared with wild-type mice [13]. An antibody-based SFRP2 blockade strategy can also reduce myocardial fibrosis, increase angiogenesis, and improve cardiac function in the failing hamster heart [15]. In contrast, other studies have shown that exogenous SFRP2 can inhibit type I procollagen maturation in primary cardiac fibroblast culture medium. Injection of SFRP2 protein into the infarct area of the rat left ventricle inhibited MI-induced fibrosis and significantly improved cardiac function [11, 12]. Furthermore, in mice with experimental autoimmune myocarditis, SFRP2 inhibited the control of myofibroblast formation and myocardial fibrosis progression by transforming growth factor-*β*-dependent Wnt secretion [12]. The inconsistencies in these results may be attributable to the use of different animal models and pathophysiological conditions. It has also been proposed that the effect of SFRP2 on myocardial fibrosis is bidirectional and concentration-dependent. Mastri et al. speculate that high doses of SFRP2 can effectively inhibit the canonical Wnt signaling in the myocardium, while producing antifibrotic effects [16]. Similarly, the work of Alfaro et al. revealed that low levels of SFRP2 promote procollagen C protease activity, which in turn promotes fibrosis, whereas high concentrations of SFRP2 have the opposite effect [29]. In our recently published study, we reported that in rat with acute myocardial infarction, transplantation of bone marrow mesenchymal stem cell overexpression insulin-like growth factor-1 greatly reduced infarct volume and myocardial fibrosis. These effects were mediated by the expression of SFRP2 [30]. Therefore, we propose that the association of SFRP2 and increased ECV fraction could be reflective of SFRP2 acting as a compensatory factor (rather than a risk factor) to counteract myocardial fibrosis during the development of HF. This proposal takes into consideration the ex vivo studies showing that SFRP2 may exert multiple other protective roles in different pathophysiological processes of cardiovascular disease, including inducing angiogenesis [31] and inhibiting cardiomyocyte apoptosis [32–34]. Furthermore, our results showed that an increased level of SFRP2 was not associated with prognosis in Cox regression analysis, which also supports our notion that SFRP2 is a marker, but not a risk factor, of myocardial fibrosis.

It is well documented that diabetes is an important risk factor associated with myocardial fibrosis. In linear correlation analysis, we also found that HbA1c was positively associated with ECV fraction, consistent with previous studies [6, 28, 35]. In addition, we found that hs-CRP was correlated with ECV fraction. This was unsurprising because HF is considered, in part, a low-grade inflammatory disorder, and inflammation is believed to contribute to HF progression [36]. However, in multivariable regression analysis, no significant associations were observed among HbA1c, hs-CRP, and ECV fraction. This may have been due to the limited study sample size. The main strengths of our study are its prospective design and the inclusion of high-risk HF patients, which allowed us to explore the association of ECV fraction and prognosis. Furthermore, we collected and adjusted multiple cardiovascular risk factors. However, some limitations should be noted. First, due to its high cost and time-consuming nature of CMR, our study had a relatively small sample size and had short-term duration of follow-up. Therefore, these results should be interpreted with caution and need to be verified in large-sample studies. Second, patients with HFpEF may carry distinct different pathophysiological characteristics with those with HFrEF. Unfortunately, our study had limited statistical power to calculate the association of SFRP2 and ECV fraction in HF patients with different EF ranges, respectively. Third, the level of SFRP2 was only measured at baseline, not during follow-up. Thus, any changes in SFRP2 level that may have occurred in response to treatment of HF are unknown and require further exploration. Fourth, we did not investigate other members of the SFRP family, such as SFRP3, which is reportedly associated with prognosis in HF patients [37, 38]. Although SFRP2 and SFRP3 share a similar structure, they may exert different effects on HF. It will be interesting to further examine the potentially unique roles of SFRP2 and SFRP3 in myocardial fibrosis.

## 5. Conclusions

ECV fraction was significantly associated with the prognosis in patients with advanced HF. SFRP2 is a novel biomarker of myocardial fibrosis in HF, as measured by ECV fraction. Further studies are needed to explore the potential therapeutic value of SFRP2 in the prevention or treatment of myocardial fibrosis.

## Figures and Tables

**Table 1:** Baseline characteristics of the included HF patients.

	All HF patients (*n* = 72)	With events (*n* = 27)	Without events (*n* = 45)	*P* value
*Clinical characteristics and comorbidities*				
Age (years)	57.7 (9.2)	56.9 (8.9)	58.1 (9.5)	0.45
Male [*n* (%)]	49 (72.9)	17 (63.0)	32 (71.1)	0.65
Smoking [*n* (%)]	24 (33.3)	11 (40.7)	13 (28.9)	0.44
Hypertension [*n* (%)]	49 (68.1)	18 (66.7)	31 (68.9)	0.95
Diabetes [*n* (%)]	32 (44.4)	13 (48.1)	19 (42.2)	0.81
Atrial fibrillation/flutter [*n* (%)]	33 (45.8)	13 (48.1)	20 (44.4)	0.95
*Type of heart failure*				0.76
HFrEF [*n* (%)]	19 (16.4)	8 (29.6)	11 (24.4)	
HFmrEF [*n* (%)]	17 (23.6)	7 (25.9)	10 (22.2)	
HFpEF [*n* (%)]	36 (50.0)	12 (44.4)	24 (53.3)	
LVEF	50.0 (38.9, 59.4)	46.0 (38.9, 58.0)	52.1 (38.5, 60.1)	0.59
LVEF in patients with HFrEF	33.2 (27.9, 36.7)	32.1 (27.5, 36.5)	35.3 (28.4, 37.0)	0.74
LVEF in patients with HFmrEF	45.1 (43.2, 47.8)	44.6 (43.0, 47.5)	46.0 (43.2, 48.0)	0.67
LVEF in patients with HFpEF	56.9 (53.4, 67.2)	55.8 (53.2, 68.0)	58.2 (54.0, 66.3)	0.88
*Causes of heart failure*				
Ischemic heart disease	48 (66.7)	16 (59.3)	32 (71.1)	0.44
Valvular heart disease	14 (19.4)	7 (25.9)	7 (15.6)	0.45
Dilated cardiomyopathy	10 (13.9)	4 (14.8)	6 (13.3)	0.86
*Current medication*				
ACEI/ARBs [*n* (%)]	52 (72.2)	22 (81.5)	30 (66.7)	0.28
Aldosterone antagonist [*n* (%)]	50 (69.4)	18 (66.7)	32 (71.1)	0.89
CCB [*n* (%)]	22 (30.6)	9 (33.3)	13 (28.9)	0.89
Beta-blockers [*n* (%)]	33 (45.8)	14 (51.9)	19 (42.2)	0.58
Loop diuretics/HCT [*n* (%)]	58 (80.6)	25 (92.6)	33 (73.3)	0.09
Digoxin [*n* (%)]	42 (58.3)	15 (55.6)	27 (60.0)	0.90
Statins [*n* (%)]	59 (81.9)	24 (88.9)	35 (77.8)	0.38
Antithrombotics [*n* (%)]	55 (76.4)	19 (70.4)	36 (80.0)	0.52
*Physical examination*				
Heart rate (beats/min)	91.1 (18.5)	93.0 (17.3)	89.9 (19.3)	0.40
Systolic BP (mmHg)	147.6 (23.9)	143.3 (24.4)	150.1 (23.5)	0.25
Diastolic BP (mm Hg)	81.5 (16.5)	79.8 (16.5)	82.5 (16.7)	0.51
BMI (kg/m^2^)	25.4 (5.0)	26.2 (5.2)	25.0 (4.9)	0.32
*Laboratory indices*				
Hemoglobin (g/L)	116.9 (18.5)	115.2 (18.8)	117.8 (18.5)	0.34
ALT (IU/L)	34.9 (32.7, 44.8)	34.5 (32.5, 47.5)	36.9 (33.4, 44.8)	0.53
eGFR (mL/min/1.73 m^2^)	52.8 (45.4, 73.5)	52.7 (45.3, 73.0)	52.9 (45.4, 73.7)	0.94
FPG (mmol/L)	7.8 (6.1, 10.4)	7.4 (5.8, 10.3)	8.2 (6.5, 10.4)	0.31
HbA1c	6.4 (5.8, 7.6)	6.0 (5.7, 7.4)	6.4 (5.9, 7.8)	0.22
TC (mmol/L)	4.9 (3.9, 5.4)	4.9 (3.9, 5.3)	4.9 (3.9, 5.6)	0.67
LDL-C (mmol/L)	2.7 (2.2, 2.9)	2.6 (2.1, 2.9)	2.8 (2.4, 2.9)	0.12
HDL-C (mmol/L)	0.9 (0.9, 1.1)	0.9 (0.8, 1.1)	1.0 (0.9, 1.1)	0.38
Triglyceride (mmol/L)	2.3 (1.9, 3.2)	2.0 (1.9, 2.8)	2.5 (1.9, 3.4)	0.30
hs-CRP (mg/L)	7.6 (2.1, 9.7)	8.0 (4.0, 14.1)	5.8 (1.6, 8.9)	0.08
Sodium (mmol/L)	133.8 (11.4)	134.2 (11.8)	133.5 (11.3)	0.80
Potassium (mmol/L)	4.1 (0.9)	4.0 (0.9)	4.3 (0.8)	0.22
NT-proBNP (ng/L)	4251.3 (1757.6, 8957.8.1)	6942.9 (5410.2, 10199.5)	3763.6 (1965.6, 8495.5.5)	0.03
SFRP2 (*μ*g/L)	30.5 (23.3, 33.4)	32.0 (27.8, 35.9)	28.1 (21.1, 33.2)	0.04
ECV fraction (%)	35.6 (32.1-40.6)	39.2 (34.7-41.4)	33.3 (30.7-37.0)	0.01

Continuous variables are presented as median (interquartile range) or mean (standard deviation). Categorical variables are expressed as number (percentages). ACEI/ARB: angiotensin-converting enzyme inhibitors or angiotensin II receptor blockers; ALT: alanine aminotransferase; BMI: body mass index; BP: blood pressure; CCB: calcium channel blocker; ECV: extracellular volume; eGFR: estimated glomerular filtration rate; FPG: fasting plasma glucose; HbA1c: glycated hemoglobin; HCT: hydrochlorothiazide; HDL-C: high-density lipoprotein cholesterol; HF: heart failure; HFmEF: HF with midrange ejection fraction; HFpEF: HF with preserved ejection fraction; HFrEF: HF with reduced ejection fraction; hs-CRP: high-sensitivity C-reactive protein; LDL-C: low-density lipoprotein cholesterol; LVEF: left ventricular ejection fraction; NT-proBNP: N-terminal pro-B-type natriuretic peptide; SFRP2: secreted frizzled-related protein 2.

**Table 2:** Clinical characteristics of the patients at discharge.

	All HF patients (*n* = 72)	With events (*n* = 27)	Without events (*n* = 45)	*P* value
*Physical examination*				
Heart rate (beats/min)	80.3 (9.3)	82.3 (9.1)	79.2 (9.9)	0.19
Systolic BP (mmHg)	133.5 (16.6)	136.8 (20.8)	131.5 (13.3)	0.19
Diastolic BP (mmHg)	77.3 (13.6)	76.5 (13.6)	77.8 (13.8)	0.70
*Medication at discharge*				
ACEI/ARBs [*n* (%)]	56 (77.8)	22 (81.5)	34 (75.6)	0.77
Aldosterone antagonist [*n* (%)]	52 (72.2)	20 (74.1)	32 (71.1)	0.96
CCB [*n* (%)]	29 (40.3)	12 (44.4)	17 (37.8)	0.76
Beta-blockers [*n* (%)]	43 (59.7)	18 (66.7)	25 (55.6)	0.49
Loop diuretics/HCT [*n* (%)]	58 (80.6)	25 (92.6)	33 (73.3)	0.09
Digoxin [*n* (%)]	37 (51.4)	13 (48.1)	24 (53.3)	0.86
Statins [*n* (%)]	60 (83.3)	24 (88.9)	36 (80.0)	0.51
Antithrombotics [*n* (%)]	58 (80.6)	21 (77.8)	37 (82.2)	0.88

Continuous variables are presented as median (interquartile range) or mean (standard deviation). Categorical variables are expressed as number (percentages). ACEI/ARB: angiotensin-converting enzyme inhibitors or angiotensin II receptor blockers; BP: blood pressure; CCB: calcium channel blocker; HCT: hydrochlorothiazide.

**Table 3:** Association between ECV fraction and clinical variables in HF patients.

Variables	*Rho*	*P* value
Age	-0.12	0.43
Sex	0.16	0.28
Smoking	0.07	0.67
Ischemic aetiology	-0.18	0.26
Hypertension	0.16	0.27
Atrial fibrillation/flutter	0.04	0.77
log_2_ (LVEF)	-0.11	0.46
Heart rate	0.06	0.69
Systolic BP	-0.23	0.11
BMI	0.11	0.47
Hemoglobin	0.01	0.92
log_2_ (ALT)	0.04	0.79
log_2_ (eGFR)	0.03	0.85
FPG	-0.02	0.90
HbA1c	0.29	0.047
TC	-0.22	0.12
HDL-C	0.01	0.93
log_2_ (triglyceride)	-0.04	0.78
log_2_ (hs-CRP)	0.31	0.02
Sodium	-0.06	0.67
Potassium	-0.20	0.17
log_2_ (NT-proBNP)	0.06	0.69
log_2_ (SFRP2)	0.33	0.02

BMI: body mass index; BP: blood pressure; ALT: alanine aminotransferase; ECV: extracellular volume; eGFR: estimated glomerular filtration rate; FPG: fasting plasma glucose; HbA1c: glycated hemoglobin; HDL-C: high-density lipoprotein cholesterol; HF: heart failure; hs-CRP: high-sensitivity C-reactive protein; LVEF: left ventricular ejection fraction; NT-proBNP: N-terminal pro-B-type natriuretic peptide; SFRP2: secreted frizzled-related protein 2.

**Table 4:** Prognostic value of clinical variables in Cox regression models.

Variables	Univariate		Multivariate	
	HR (95% CI)	*P* value	HR (95% CI)	*P* value
Age (each 10 years)	1.59 (1.19, 2.12)	0.001	1.13 (1.04, 1.22)	0.002
Sex (female vs. male)	1.16 (0.36, 3.76)	0.80		
BMI (each 1 kg/m^2^)	1.09 (0.97, 1.64)	0.27		
Diabetes (yes vs. no)	1.26 (0.97, 1.64)	0.08	1.09 (0.90, 1.32)	0.38
Smoking (yes vs. no)	1.42 (0.39, 5.14)	0.59		
SBP (each 10 mmHg)	1.00 (0.98, 1.03)	0.85		
NT-proBNP (each doubling)	3.46 (1.39, 8.61)	0.008	2.46 (1.05, 5.76)	0.04
hs-CRP (each doubling)	0.52 (0.23, 1.20)	0.13		
Sfrp2 (each doubling)	1.25 (0.97, 1.64)	0.09	1.18 (0.75, 1.86)	0.47
ECV fraction (each doubling)	1.79 (1.10, 2.91)	0.02	1.68 (1.03, 2.74)	0.04
LVEF (each doubling)	0.49 (0.18, 1.33)	0.16		
eGFR (each doubling)	0.42 (0.07, 2.42)	0.33		

BMI: body mass index; CI: confidence interval; ECV: extracellular volume; eGFR: estimated glomerular filtration rate; HR: hazard ratio; LVEF: left ventricular ejection fraction; SBP: systolic blood pressure; hs-CRP: high-sensitivity C-reactive protein; NT-proBNP: N-terminal pro-B-type natriuretic peptide; SFRP2: secreted frizzled-related protein 2.

## Data Availability

The research data used to support the findings of this study are currently under embargo. Requests for data, 12 months after publication of this article, will be considered by the corresponding author, Dr. Yuli Huang, Department of Cardiology, Shunde Hospital, Southern Medical University, Penglai Road, Daliang Town, Shunde District, Foshan 528300, China. E-mail: hyuli821@smu.edu.cn or hyuli821@163.com.
